# Research disciplinary interactions on scientific collaboration network in photocatalytic hydrogen evolution: Characteristics and dynamics

**DOI:** 10.1371/journal.pone.0266404

**Published:** 2022-04-14

**Authors:** Xiaojie Yao, Yuan Hu, Xiaomin Zou, Wenjian Qu

**Affiliations:** School of Public Policy and Administration, Nanchang University, Nanchang, Jiangxi, The People’s Republic of China; Zewail City of Science and Technology, EGYPT

## Abstract

Interdisciplinary scientific collaboration promotes the innovative development of scientific research. Photocatalytic hydrogen evolution (PHE) is a typical interdisciplinary subject. This study aims to explore the characteristics of discipline interaction and the temporal evolution in the field. Bibliometric analysis could be used to understand the stage of research in a particular subject. In this work, the publications on the topic in Web of Science (WoS) platform from 1999 to 2020 were selected. On the basis of social network theory, the characteristics of interdisciplinary were revealed from three perspectives. First, the disciplinary interaction network is constructed through disciplinary co-occurrence to detect the characteristics of interaction structure among different disciplines. Then the node centrality index is employed to explore the influence of disciplines in the interactive network by using network centrality analysis. Moreover, the dynamic of discipline interaction evolution is studied using blockmodeling analysis. In the field of PHE, the number of disciplines and the intensity of interaction among different subjects gradually increased in the past 20 years. Chemistry and Material Sciences are the core discipline, and they play an important role in the network. The whole network is divided into different discipline groups. The scale of the discipline group is becoming large, and the disciplinary interaction is becoming more complex. The obtained results are helpful for guiding scholars to carry out interdisciplinary interaction. The methods of detecting interdisciplinary interactive relationship could provide paths for interdisciplinary research in other fields.

## 1 Introduction

Facing the advent of the era of big science, the global competition of science and technology begins to focus on scientific collaboration [1]. The trend of collaboration and network development [2] is becoming increasingly remarkable, and the number of related publications show an explosive growth [3]. Scientific collaboration promotes communication among various academic information and knowledge, and speeds up the spread of knowledge [4]. In the information age, scientific studies are breaking the limitation of single subject knowledge and integrating different disciplines to achieve scientific innovation [5]. Interdisciplinary collaboration plays a key role in the realization of original scientific research [6]. With the trend of disciplinary integration and penetration, the phenomenon of interdisciplinary scientific collaboration among scholars is becoming increasingly common.

The interactive relationship among scholars is an important embodiment in the scientific collaboration network [7]. Interaction relationship is also formed among various disciplines in interdisciplinary scientific research [8]. Researchers have different interactive characteristics in different disciplines [9]. In new materials science, photocatalysis technology is a core pursuit to mankind solving global energy shortage and environmental pollution. The important breakthroughs about photocatalytic hydrogen evolution (PHE) are generally created by the cooperation of different disciplines rather than a single traditional discipline, such as chemistry or material. However, the study of interdisciplinary collaboration network in the scientific field of PHE is rarely reported. Whether researchers have interdisciplinary scientific collaboration and how the characteristics and dynamics of disciplinary interactions in the field over the past 20 years remain unclear. Therefore, interdisciplinary network research was conducted to explore its interdisciplinary status in the field and help with scientific development.

This study focused on empirical research on the scientific field of PHE, which has not been reported at present. This study aimed to reveal the current situation of interdisciplinary on scientific collaboration in the field and examine the interdisciplinary relationship characteristics and evolution. Three research questions were solved:

Question 1: What are the distribution of different disciplines and the trend characteristics in the scientific collaboration of PHE?
Question 2: What are the disciplinary interactive relationship characteristics and the key disciplines in the scientific field?
Question 3: What are the dynamics of relationship among different disciplines over the past 20 years?

On the basis of social network theory, publications in the ISI Web of Science (WoS) database regarding the scientific field of PHE over the past 20 years were selected to answer the above questions. Bibliometrics and co-occurrence analysis methods were used. WoS subject categories were taken as the discipline measurement index to explore the interdisciplinary trend and characters in the field.

## 2 Literature review

### 2.1 Interdisciplinary scientific collaboration

Scientific collaboration can establish academic research information communication networks among researchers [10]. The collaboration network promotes researchers to carry out various academic information and knowledge [1], and then knowledge can be widely spread. Subsequently, various informal academic information interaction and cooperation are formed in academic social networks [11]. Scientific collaboration can appear in different scholars, disciplines, institutions, regions or countries [12, 13].

Interdisciplinary collaboration and communication among different disciplines becomes urgent in the field of scientific research with the trend of disciplinary integration and mutual penetration [14]. Interdisciplinary collaboration has become synonymous with progression of research due to the scientific complexity of problems currently under study [15]. Interdisciplinary has a positive effect on knowledge acquisition [16], and it effectively promotes scientific cooperation [17]. Compared with individual research, collaboration among scholars can promote the flow of knowledge among different disciplines [18]. Interdisciplinary research (IDR) integrates multidisciplinary data, methods, tools, concepts, and theories to solve problems that could not be solved by a single discipline or field of research practice [19]. IDR uses cross-thinking to break the barriers among different disciplines and realize the innovative development of science and technology [20]. Inquiring about the different forms, structure and modes of collaboration can help researchers further understand the distribution of existing research forces and the internal changes of the disciplines in a field [21]. Previous studies have explored interdisciplinary measurement and the design of measurement methods. Some measurement indexes are constructed [22].

Interdisciplinary scientific collaborations are studied in several manners: by citation analysis [23, 24], by periodical analysis [25], and by subject topic analysis [26]. Collaboration also exists in cross-country [27] and co-authorship [28]. Previous studies explored the effect and internal motivation of international scientific research collaboration [29]. Institutional collaboration focused on the distribution of institutions, the publication of the institutions, and the mode of collaboration network [30]. In particular, the author analysis is more commonly used to explore scientific research cooperation because it is very simple with objective means to measure scientific collaboration [31]. A co-author network was constructed to obtain authors with high centrality and activity in a certain field and explore the relationship among authors [32]. Some studies also explored the network distribution of the cooperation theme and the sub-network model [33]. Existing research mainly used bibliometric analysis to discuss the distribution of disciplines, the changing trend of the number of collaboration papers, and the structure mode of collaboration network.

The research of interdisciplinary measurement mainly adopts bibliometric method.

Bibliometric analysis is conducted to identify the research trend and interests in a field. Bibliometrics can explore the research structure, characteristics and laws in a field through the movement and change law of literature itself [34]. The method can effectively evaluate and predict the research status and development trend in this field with statistics and analysis of research results [35].

### 2.2 Interdisciplinary relationship

Research on interdisciplinary relationship has become an interesting direction for more scholars [16, 36]. More studies tend to carry out citation relationship, co-word analysis and cluster analysis to explore the relationship among disciplines. Based on co-word analysis, the relationship could be constructed among different disciplines in a certain research field [23]. Clustering analysis can classify data sources into different groups by clustering algorithm on the basis of similarity [37]. Cluster analysis technology can effectively analyze the cross relationship between disciplines. Small et al. used clustering method to study the similarity and intersection of multi disciplines on the basis of citation analysis [36].

Mapping the relationships of the experts, fields and problems in science, has been widely used in academic research [38, 39]. Social network analysis (SNA) focuses on the relations and ties between actors, and it could achieve quantitative description and measurement [40]. It is a good method to examine interdisciplinary relationship. In reported studies, the interdisciplinary relationships of collaboration network were discussed through the collaboration of scholars, organizations, regions or countries [41]. For example, Quintella et al. examined the relations between the scientific institutional, structural, and relational characteristics of inter-institutional and intra-institutional scientific knowledge networks [42].

The existing studies, whether based on citation analysis or keyword analysis, focus on interdisciplinary exploration, and ignore the research on the comprehensive evolution and development of multidisciplinary in a certain research field.

## 3 Data and methods

### 3.1 Data and samples

The WoS database is the most comprehensive academic information resource covering the largest number of disciplines. The database contains various core academic journals with the most influence in natural science, engineering technology, biomedical and other research fields. The publications in WoS are classified into different subject categories, containing 18,000 journals and 254 subject categories. In this study, the given WoS subject categories that represent the disciplinary of the document were obtained. Data were collected from the WoS platform, and documents from the field of academic research about PHE were retrieved. The search limits are the following:

- Database: WoS Core Collection
- Search Expression: (TS = Photocataly* and Hydrogen)
- Year Published: (PY = 1999 OR PY = 2000 OR … OR PY = 2020)
- Document Type: (DT = Article)
- Collection Citation Indexes: SCI-Expanded
- Retrieval Time: February 16, 2021

The time-span of the data from 1999 to 2020 was chosen, which could better reflect the general situation and evolution of the research field. The data were mainly restricted in SCI-Expanded because they are the most prestigious journals, and they provide robust and frequently used sources for bibliometric research. The general search resulted in 29,989 items, wherein 27,741 articles were published in journals. A reduced part of the production (1.30%) was found in regional records (nonstandard or duplicate records). Finally, 27,380 publications were collected, and then the downloaded data were structured and stored in the database for analysis.

### 3.2 Methods

Social network analysis (SNA) is a new research thought used in sociology, psychology, communication science and other fields since 1970s, which is a quantitative analysis method developed on the basis of mathematics and graph theory [43]. Given that SNA has the advantage of measuring and visualizing the structure of social relationships [40], it was chosen to investigate the interactive relationships in the research field. Thus, the implicit interactive relationship among disciplines could be well revealed by constructing a discipline network and visualizing the interaction relationship on basis of SNA.

Meanwhile, SNA could explore the social network relationship from many different perspectives. In this study, (1) co-occurrence analysis, (2) node centrality analysis, and (3) blockmodeling were employed to establish a new concept of discipline relationship.

#### 3.2.1 Co-occurrence analysis

Co-occurrence analysis is a quantitative method used to study the co-occurrence characteristics in literatures, such as title, author, keyword, and organization [44]. As a means of text knowledge mining, co-occurrence cold well reveal the content relevance of information and the knowledge implied in literature [45]. The correlation between the common features and the degree of correlation were measured by co-occurrence frequency [46]. The key to co-occurrence analysis is to construct a co-occurrence matrix of the disciplines, and then the network could be formed by this co-occurrence matrix.

Moreover, in disciplinary network, WoS subject categories are used to represent different disciplines. Categories refer to the classification of the title field of a publication in the database. Thus, WoS categories based on the database platform could characterize different disciplines [47]. Many studies used WoS subject categories as the discipline index to measure interdisciplinary characteristics [48]. When a publication contains two or more subject categories at the same time, a relevance occurs among these disciplines. When the frequency of common occurrence is higher, the relevance is stronger [49].

Therefore, co-occurrence analysis of WoS categories could be used to construct the disciplinary interaction network and measure the structural interactive characteristics in the field of PHE. The WoS categories, the corresponding frequency, and the co-occurrence matrix, were extracted by Bibexcel software [50].

#### 3.2.2 Node degree analysis

Network node degree analysis was carried out to detect the disciplinary influence in interdisciplinary structure network. Node degree distribution is an important structural character of networks [51], it could examine the centrality of a node in the whole network and judge the importance of the structural centrality for networks [52]. The measurement of node centrality could well identify the influence and role in the network.

The centrality indicators include (a) degree centrality, (b) betweenness centrality and (c) closeness centrality. Degree centrality reflects the direct influence of one node to the other in the interaction network of scientific collaboration [53]. Betweenness centrality reflects the social status of individuals. Disciplines with high betweenness centrality often play a bridge and intermediary role in the interactive collaboration of different disciplines. The higher the degree of betweenness center is, the more it plays the role of intermediary bridge in the network. [54, 55]. Closeness centrality is measured by the shortest distance between nodes and other nodes, and it evaluates the importance of nodes through network structure. The greater the closeness centrality of nodes is, the more important they are in the network [51]. When the centrality of a node is large, it suggests that the node has a great influence in the network [56].

The discipline with high influence in the network has closer relations with other disciplines, and it identifies the key discipline in this field [53]. From the position and role of the disciplines, the characteristics of interactive relationships were explored. Centrality index was employed to measure the key disciplines in the interdisciplinary structure network in the field of PHE. On basis of centrality degree analysis, the node degree value of each discipline was measured by Ucinet 6.0 [57].

#### 3.2.3 Blockmodeling analysis

Blockmodeling is a typical quantitative research on social network that is based on the theory of differential order pattern, and it could simplify a big network into a small structure [58]. It could also examine the interaction and collaboration relationship within and among the structure status in network through the flow of knowledge [59]. Two basic procedures are used to construct the blockmodeling of interdisciplinary networks. First, the network is divided into small disciplinary groups. Through blockmodeling, the overall network structure and relationship mode are partitioned by comparing the network density of each sub-group with that of the whole network [60]. Blockmodeling includes several approaches to examine network location model [59]. Here, Concor algorithm was used to divide different blocks. Then, all the different disciplines were divided into different modules, thus forming different disciplinary groups. Second, disciplinary interactive relationship was examined inter-block and intra-block. The modules include 1-block or 0-block, which could be determined through a comparison of each block density with the whole network density. 1-Block, which presents the density greater than the whole network, is the opposite of 0-block. The subject knowledge in 1-block flows to 0-block, and forms scientific interactive collaboration. The density of each block could be obtained via blockmodeling procedure on Ucinet 6.0.

Considering that a discipline interactive network is a valued network, blockmodeling analysis was carried out to observe the dynamics of discipline relationship by examining the interactions among inter-disciplinary and intra-disciplinary groups from 1999 to 2000. By observing the temporal evolution, the dynamics of the discipline cluster and the interactive relationships between the groups could be obtained.

## 4 Results

### 4.1 Disciplinary distribution

**Fig 1:**
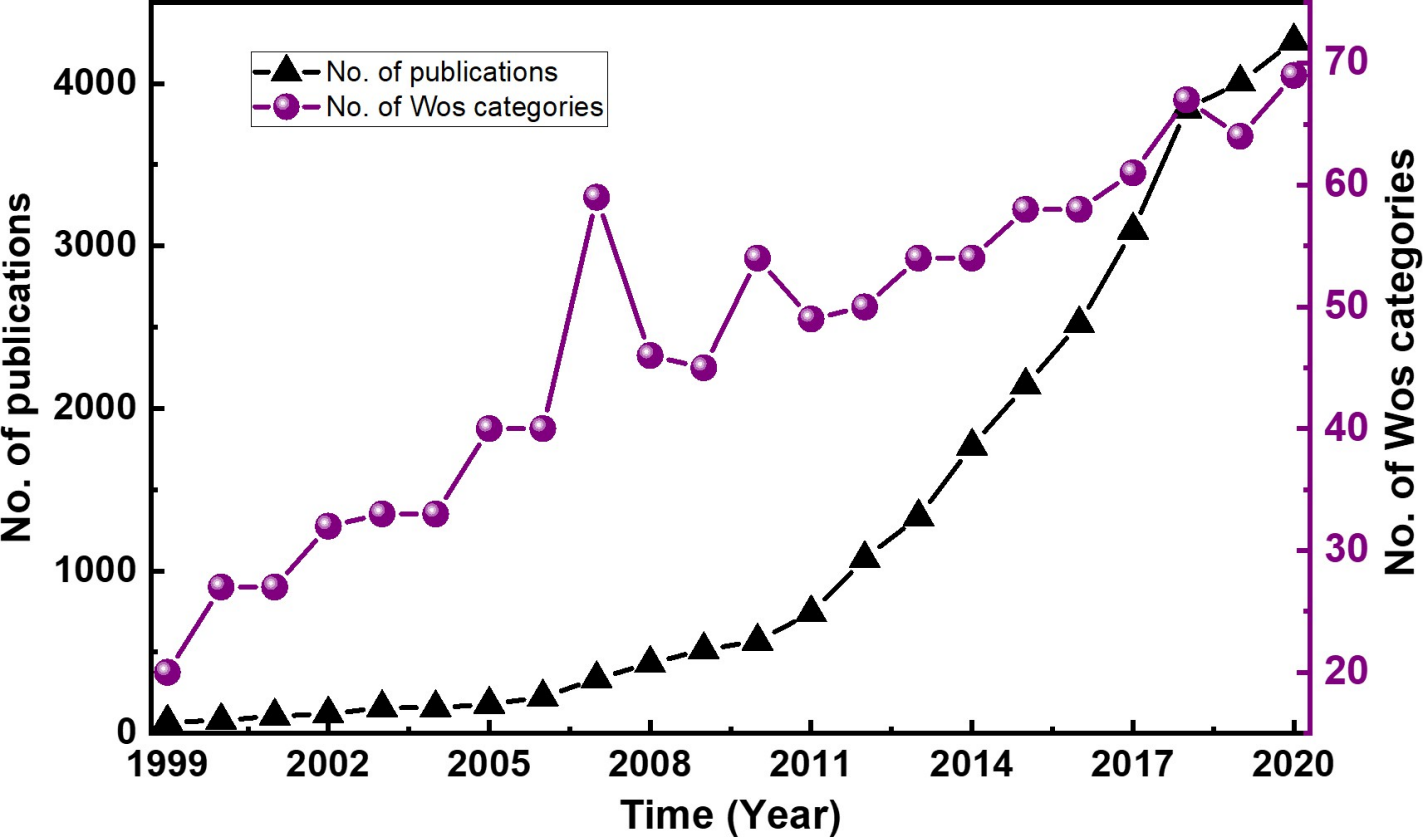
Distributions of the publication years in photocatalytic research. The black line represents the number of published studies, and the purple line represents the number of WoS subject categories.

The number of subject categories in each year were counted to investigate the discipline distribution of time dimension. The statistics of the annual distribution of publications were also employed to understand the overall research status. Finally, 27,380 publications and 99 WoS categories were collected in 1999–2020. Fig 1 presents the temporal evolution of publications and discipline categories and its obsolescence. From 1999 to 2020, the number of publications substantially increased, and the WoS subject categories increased practically in a constant manner. In detail, the growth rate of publications sharply increased after 2010. Meanwhile, the disciplines also maintained a steady growth, suggesting that the research of PHE attracted increasing attention form different disciplines.

The total number of publications of each discipline was analyzed to explore the horizontal distribution of disciplines. The number of publications of the top 10 disciplines and the corresponding proportion in all publications are shown in Table 1. The top three disciplines were Chemistry Physical (12,567, accounting for 45.9%), Materials Science Multidisciplinary (7,255, accounting for 26.5%) and Chemistry Multidisciplinary (6,187, accounting for 22.6%). Among them, Chemistry Physical accounted for nearly half in all the documents.

**Table 1:** The publications of WoS subject categories (Top 10).

WoS Categories	No. of publication	%WC in publications
Chemistry Physical	12567	45.9
Materials Science Multidisciplinary	7255	26.5
Chemistry Multidisciplinary	6187	22.6
Engineering Chemical	4461	16.3
Nanoscience Nanotechnology	3504	12.8
Energy Fuels	3477	12.7
Physics Applied	3395	12.4
Engineering Environmental	2936	10.7
Physics Condensed Matter	2245	8.2
Electrochemistry	2080	7.6

**Fig 2:**
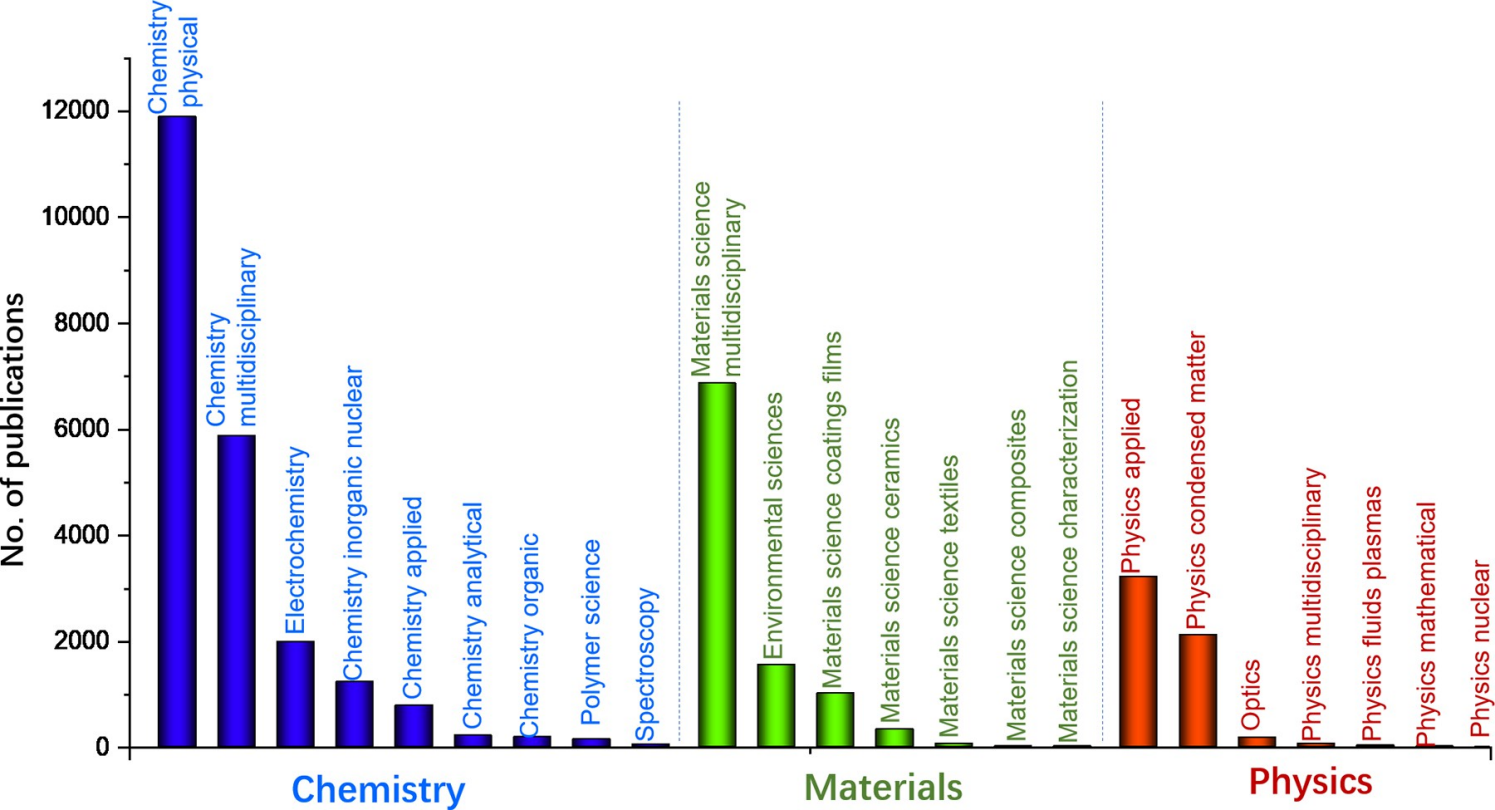
The number of papers published of the disciplines of Chemistry, Materials and Physics (Top 3 of first level discipline).

The WoS categories classified into first-level disciplines were analyzed to discover the distribution in higher-level disciplines. First-level disciplines include Chemistry, Materials Science and Engineering, Physics, Biology, Hydraulic Engineering, Medicine Science and Environmental Science and Engineering. The number of publications of on the top three first-level disciplines is shown in Fig 2. Chemistry, Materials, and Physics occupied the top three places. A large number of publications in Chemistry suggested that PHE is mainly studied in Chemistry. Among the first level discipline of Chemistry, the number was mainly distributed in Chemistry Physical, Chemistry Multidisciplinary, and Electrochemistry. Among the first-level discipline of Materials, the publications were mainly in Materials Science Multidisciplinary, and the number was substantially more than that of other disciplines. Among the Physics discipline, the publications were mainly from Physics Applied and Physics Condensed Matter. The above analysis indicated that the studies of PHE attracted high academic attentions in the fields of Chemistry, Materials Science, and Physics. The finding suggested that the research of PHE first appeared in the above fields and gave rise to the interaction among another.

### 4.2 Network analysis of disciplines

**Fig 3:**
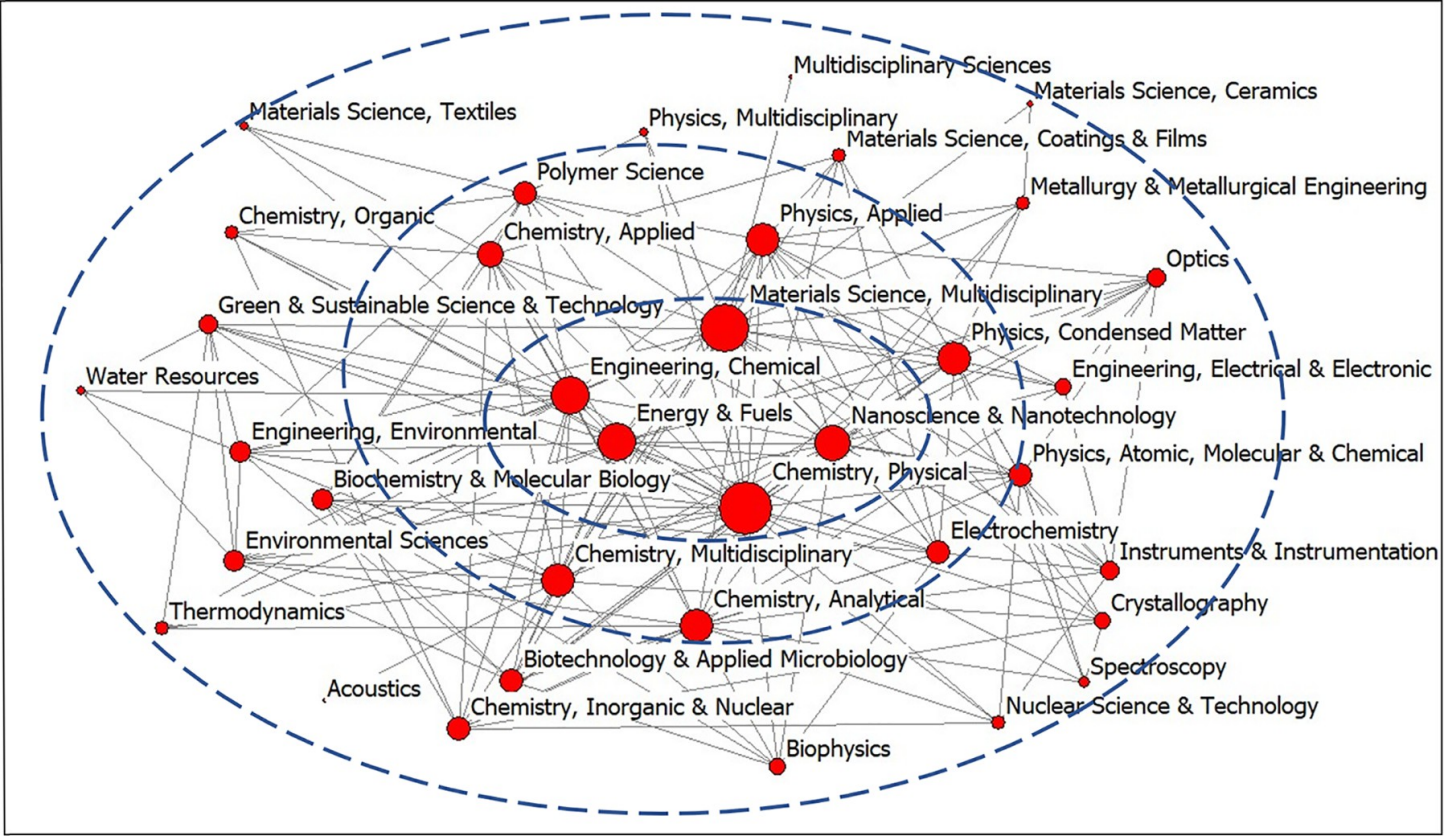
The disciplinary interactive network in the period of 1999–2020.

Word frequency analysis is an important means of text mining by counting and analyzing the number of important words in the text. The law of hot spots or the change trend can be found. In this study, the frequency of WoS subject categories in the publications of PHE were analyzed. The co-occurrence matrix of WoS categories in the period of 1999–2020 was constructed and visualized by Ucinet 6.0, as shown in Fig 3. In the network, the nodes represented the discipline, and the links represented the discipline connections [61]. The network in a single year was constructed by subject categories with co-occurrence frequency more than 3 times. Different from a single year, the total network from 1999 to 2020 was constructed by the co-occurrence frequency more than 40 times to obtain clear network characteristics. In the period of 1999–2020, the disciplinaries of Materials Science Multidisciplinary, Chemistry Physical, Physics Applied and Nanoscience Nanotechnology are in the core position of the interactive network, suggesting that their interaction relationship is closer to other disciplines in the network. The disciplinaries in the center have more frequent cooperative communication, showing stronger relationship, whereas others in the periphery of the network have less connections and are in a weak relationship position [62].

**Fig 4:**
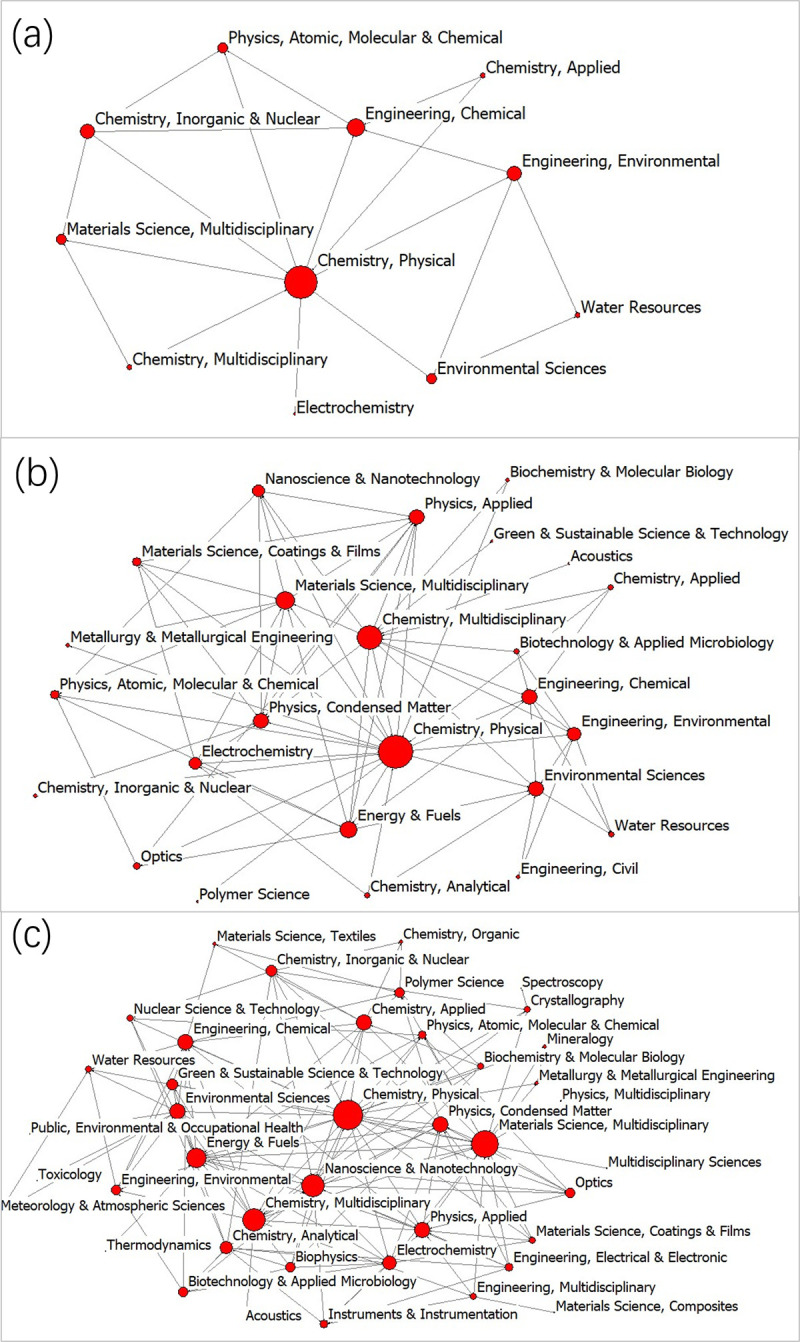
The disciplinary interactive network in 1999 (a), 2010 (b) and 2020 (c).

The movement of the overall disciplinary interactive networks was mapped. The networks in 1999, 2010 and 2020 are shown in Fig 4A–4C, respectively. As shown in Fig 4A, the network relationship was simple in 1999, where only Chemistry Physics had relationship with other subjects. However, in 2020, the network structure became complex, as shown in the Fig 4C, suggesting that the disciplinary interactive network was weak in the early years. With the deepening of research, strong interactive characteristics began to appear.

Furthermore, the structure characteristics of interactive network and the evolution were studied in detail and their corresponding network structure indicators were collected (Table 2). In 19999–2020, the number of interactive disciplines was 36 (the frequency was more than 40, forming 185 interactive links). The overall network density in 1999–2020 was 0.147, which is a relatively low level.

**Table 2:** The structure characteristics of disciplinary interaction networks.

Network indicators	1999–2020	1999	2005	2010	2015	2020
No. of discipline interaction	36	11	17	25	37	44
No. of links	185	19	45	70	125	162
Mean distance	1.692	1.568	1.698	1.717	1.947	1.972
Density	0.147	0.173	0.166	0.117	0.094	0.097
Network centralization	0.510	0.680	0.612	0.610	0.360	0.389
Clustering coefficient	0.326	0.361	0.369	0.360	0.295	0.297

In 1999, the interactive collaboration network had 11 disciplines and 19 links in the field of PHE. By 2005, the diversity of subjects increased slightly, whereas the number of interactive increased rapidly. The number of links grew faster than the disciplines, suggesting that the interaction between different disciplines gradually strengthened. After 2010, the disciplinary interactive network expanded significantly. The diversity of disciplines and the number of interactive links increased significantly. In 2015, the number of disciplines extensively increased from 25 in 2010 to 37. The decrease in clustering coefficient suggested that the relationship of disciplines moved closer to each other. In 2020, the number of disciplines was the largest, and number of links reached the top in the previous years, indicating that the interactive relation was the highest in 2020.

Therefore, in the past 20 years, the cooperation relationships in PHE gradually increased. The number of interactive disciplines also increased, and more disciplines played a role in the interactive network.

### 4.3 Centrality analysis of disciplinary influence

The degrees of network node of each discipline, including degree centrality, betweenness centrality, and closeness centrality, were collected to explore the influence of each discipline in the disciplinary interactive network in the whole period of 1999–2020 (Table 3). The node degree of each discipline in 1999 and 2020 were measured to examine the evolution trend of key disciplines, as shown in Table 4.

**Table 3:** The centrality of the main discipline in the period of 1999–2020.

First level discipline	WoS Categories	1999–2020			
		Fre.	Deg.	Bet.	Clo.
Chemistry	Spectroscopy	34.00	5.00	0.00	12.77
	Polymer Science	33.00	11.00	0.00	17.68
	Electrochemistry	12.00	12.00	12.78	3.82
	Chemistry Physical	10.00	27.00	55.95	3.70
	Chemistry Organic	9.00	6.00	0.70	3.31
	Chemistry Multidisciplinary	8.00	17.00	34.96	3.33
	Chemistry Inorganic Nuclear	7.00	12.00	5.42	3.22
	Chemistry Applied	6.00	13.00	5.69	3.12
	Chemistry Analytical	5.00	16.00	15.18	3.03
Material Science	Materials Science Multidisciplinary	22.00	25.00	83.02	6.60
	Materials Science Coatings Films	21.00	6.00	0.29	3.96
	Environmental Sciences	17.00	10.00	3.84	4.51
Physics	Physics Multidisciplinary	32.00	3.00	0.00	6.84
	Physics Condensed Matter	31.00	16.00	15.98	11.91
	Physics atomic molecular chemical	30.00	11.00	9.62	9.51
	Physics Applied	29.00	16.00	5.13	8.84
	Optics	28.00	9.00	0.45	7.97

**Table 4:** The centrality of the main discipline in 1999 and 2020.

First level discipline	WoS Categories	1999				2020			
		Fre.	Deg.	Bet.	Clo.	Fre.	Deg.	Bet.	Clo.
Chemistry	Spectroscopy	0.00	0.00	0.00	0.00	38.00	1.00	0.00	3.19
	Polymer Science	0.00	0.00	0.00	0.00	36.00	7.00	0.00	7.31
	Electrochemistry	5.00	1.00	0.00	0.00	12.00	10.00	11.77	14.09
	Chemistry Physical	4.00	9.00	15.50	12.50	10.00	22.00	57.91	3.02
	Chemistry Organic	0.00	0.00	0.00	0.00	9.00	3.00	0.63	2.85
	Chemistry Multidisciplinary	3.00	2.00	0.00	14.09	8.00	17.00	62.91	2.78
	Chemistry Inorganic Nuclear	2.00	4.00	0.00	9.09	7.00	8.00	11.17	2.77
	Chemistry Applied	1.00	2.00	0.00	9.09	6.00	11.00	5.15	2.50
	Chemistry Analytical	0.00	0.00	0.00	0.00	5.00	9.00	11.72	2.63
Material Science	Materials Science Multidisciplinary	9.00	3.00	0.00	14.09	23.00	20.00	98.44	4.70
	Materials Science Coatings Films	0.00	0.00	0.00	0.00	21.00	5.00	0.20	4.85
	Environmental Sciences	8.00	3.00	2.00	15.87	18.00	12.00	70.50	3.82
Physics	Physics Multidisciplinary	5.00	0.00	0.00	0.00	35.00	1.00	0.00	4.81
	Physics Condensed Matter	0.00	0.00	3.00	0.00	34.00	12.00	9.03	6.11
	Physics atomic molecular chemical	10.00	3.00	0.00	18.87	33.00	6.00	0.00	5.41
	Physics Applied	0.00	0.00	0.00	0.00	32.00	12.00	4.00	5.77
	Optics	0.00	0.00	0.00	0.00	31.00	7.00	0.00	4.88

In the past 20 years, the whole degree centrality of Chemistry Physical, Materials Science Multidisciplinary, and Chemistry Multidisciplinary occupied the top three places, and their corresponding values were 27, 25, and 17, respectively. This result suggested that the ability of direct interaction and connection of the three disciplines was the strongest. In terms of temporal evolution, the degree centrality of Chemistry Physical was the largest, whether in 1999 or in 2020, and the increase was remarkable from 9 to 22. This finding indicated that Chemistry Physical has always been a key discipline in the field and its influence in the network increased gradually in the process of interaction.

Betweenness centrality was used to measure the degree of control of other nodes in the network, which plays the role of bridge to communicate with other nodes. In Table 3, the betweenness centrality of Materials Science Multidisciplinary (83.08) and Chemistry Physical (55.95) was the largest in the network. The influence of Chemistry Physical was stronger in the network. In Table 4, the betweenness centrality of Chemistry Physical was still the largest in 1999 at 15.5. Meanwhile, the betweenness centrality of Materials Science Multidisciplinary was the largest in 2020 at 98.44. This change indicated that Materials Science Multidisciplinary began to dominate in 2020. However, the frequency was relatively low compared with that of Physics Atomic Molecular Chemical, Materials Science Multidisciplinary, and Environmental Sciences, suggesting that the number of Chemistry Physical decreased, but its influence in the interactive network remained great.

The closeness centrality of Polymer Science and Spectroscopy was relatively large, as shown in Table 3, indicating that they could exert influence to the other discipline nodes through network structure. The closeness centrality of Water Resources, Physics Atomic Molecular and Chemical, and Environmental Sciences was also relatively large. In 1999, the closeness centrality of Physics Atomic Molecular Chemical was 18.87, which was the largest, suggesting that this discipline could exert influence to the other disciplines through network structure. However, the closeness centrality of Electrochemistry in 2020 was 14.09, which became the largest in the network.

Great changes could be observed in the disciplines in Table 4. The frequencies of disciplines, such as Spectroscopy, Polymer Science, Chemistry Organic, Chemistry Analytical, were 0 in 1999, and they rapidly increased in 2020. In particular, those of Physics Condensed Matter and Physics Applied increased obviously from 1999 to 2020. The above results suggested that the influence of Physics improved rapidly. Meanwhile, the frequency of Spectroscopy and Physics Multidisciplinary was high, but the centrality and betweenness centrality were very small. This result indicated that academic research appeared more frequently in these disciplines, but they did not have important influence in the disciplinary interactive network.

### 4.4 Blockmodeling analysis of disciplinary interaction dynamics

On the basis of blockmodeling analysis, the dynamics of interdisciplinary interactive relationship were captured. The results of blockmodeling were presented, including the first year, 1999 (Table 5), and the last year, 2020 (Table 6). As shown in Table 5, the network was divided into seven clusters in 1999. Different clusters formed different disciplinary groups. These interactive disciplines formed seven discipline groups. Clustering divides similar objects into one group [63]. The scale of each cluster includes one or two disciplines. Different from 1999, in Table 6, eight clusters were found in 2020, and the scale of clusters became large. The scale was up to nine in 2020, whereas it was less than two in 1999.

**Table 5:** The block clustering of the disciplines in 1999.

CLUSTER 1		CLUSTER 3	
First level discipline	WoS categories	First level discipline	WoS categories
Chemistry	{Chemistry Physical}	Chemistry	{Chemistry Multidisciplinary; Chemistry Inorganic Nuclear}
CLUSTER 2		CLUSTER 4	
Chemistry	{Chemistry Applied}	Chemistry; Materials Science	{Electrochemistry; Materials Science Multidisciplinary}
CLUSTER 5		CLUSTER 7	
Chemical Engineering and Technology; Physics	{Engineering Chemical; Physics Atomic Molecular Chemical}	Hydraulic Engineering	{Water Resources}
CLUSTER 6			
Environmental Science and Engineering; Materials Science	{Engineering Environmental; Environmental Sciences}		

**Table 6:** The block clustering of the disciplines in 2020.

CLUSTER 1		CLUSTER 3	
**First level discipline**	**WoS categories**	**First level discipline**	**WoS categories**
{Physics; Bioengineering; Chemistry}	{Acoustics; Biotechnology Applied Microbiology; Chemistry Analytical}	{Chemistry; Materials Science; Materials Science}	Spectroscopy; Chemistry Organic; Materials Science Composites; Polymer Science}
CLUSTER 2		CLUSTER 4	
{Bioengineering; Biology; Chemistry}	{Biochemistry Molecular Biology; Biophysics; Chemistry Applied; Chemistry Inorganic Nuclear}	{Public Health and Preventive Medicine; Basic Medicine Sciences; Atmospheric Sciences; Hydraulic Engineering}	{Public Environmental Occupational Health; Toxicology; Meteorology Atmospheric Sciences; Water Resources}
CLUSTER 5		CLUSTER 7	
{Chemistry; Electrical Engineering; Mechanical Engineering; Engineering Technology; Crystallography}	{Chemistry Multidisciplinary; Chemistry Physical Engineering Electrical Electronic; Engineering Multidisciplinary; Green Sustainable Science Technology; Nanoscience Nanotechnology; Crystallography}	{Oil and Gas Engineering; Materials Science; Chemistry; Nuclear Science and Technology; Materials Science; Chemical Engineering and Technology; Physics; Environmental Science and Engineering}	{Energy Fuels; Materials Science Multidisciplinary; Environmental Sciences; Electrochemistry; Nuclear Science Technology; Materials Science Coatings Films; Engineering Chemical; Physics Atomic Molecular Chemical; Engineering Environmental}
CLUSTER 6		CLUSTER 8	
{Instrument Science and Technology; Power Engineering and Engineering Thermophysics}	{Instruments Instrumentation; Thermodynamics}	{Metallurgical Engineering; Physics; Geology; Multidisciplinary Sciences; Physics}	{Metallurgy Metallurgical Engineering; Physics Applied; Mineralogy; Multidisciplinary Sciences; Physics Condensed Matter; Physics Multidisciplinary}

**Fig 5:**
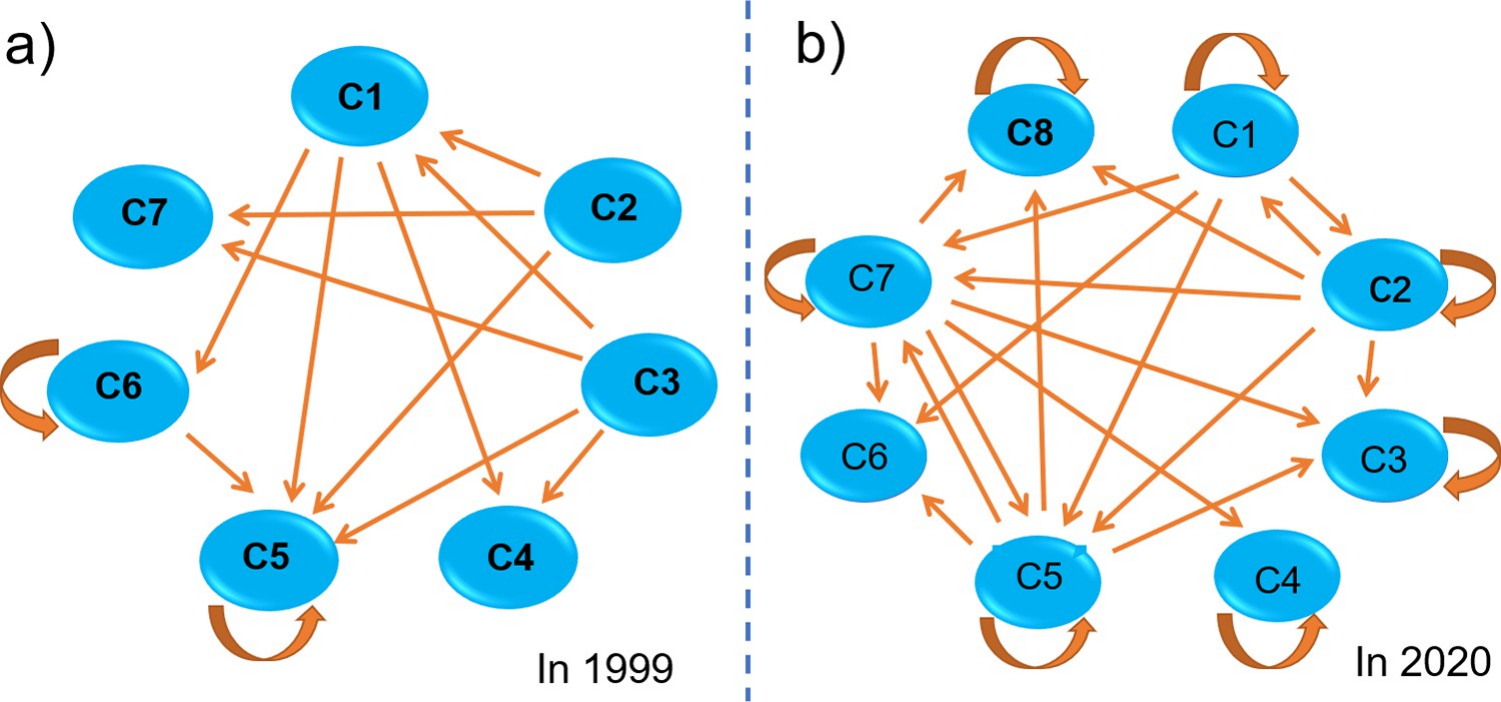
Schematic visualization of cluster modeling for 1999 (a) and 2020 (b).

The network density and the corresponding image matrix of each cluster were measured to determine the disciplinary interactive relationship between each cluster in different years. Disciplinary directional relationship inter-blocks or intra-blocks were obtained by comparing the values of network density and image matrix [64]. The visualization results of disciplinary interactive relationships about clustering models in 1999 and 2020 are shown in Fig 5, where each ellipse represented a cluster from Tables 5 and 6, and the arrow lines between ellipses represented the disciplinary interactive relationship between different clusters. The arcuate arrow indicated disciplinary interactive relationship within the cluster. In 1999, clusters 5 and 6 had a disciplinary interactive relationship, and they also interacted with the other clusters, suggesting that Chemistry and Physics, Environmental, and Materials had active interactive relationships with others. Cluster 7 was isolated in the structure position and had no interactive collaboration with the other disciplines, indicating that hydraulic engineering had no interaction with other discipline. In 2020, clusters 1, 2, 5, and 7 had strongly interactive collaboration with one another and interactive collaboration with the other clusters. Meanwhile, no isolated cluster was observed in the structure position in 2020.

## 5 Discussion

In this study, the capacity of researchers to collaborate among various disciplines to solve scientific problems in the research of PHE was observed. The structure characteristics of the disciplinary interactive network was examined. The results showed that Chemistry, Materials, and Physics Sciences were the major first-level disciplines, followed by many other disciplines, such as Biology, Hydraulic Engineering, Medicine Science, and Environmental Science. The leading role of WoS categories in Chemistry, Materials, and Physics Sciences was explored. The well-established discipline clusters and the different dynamics of interdisciplinary cooperation were also elucidated.

### 5.1 What are the characteristics of disciplinary interactive relationship and evolution

The case of disciplines was worthy of attention due to the crisscrossing and permeating between the disciplines in an emerging scientific field [65]. A total of 99 WoS categories were found in the observed years in the present study. A significant rise in the WoS categories was also found at 20 in 1999. However, in 2020, the figures increased up to 68. The disciplines of Chemistry, Materials, and Physics Sciences deserve attention as they are the major disciplines in the document, and their roles in the network change significantly over time. When considering the WoS categories, Chemistry Physical (45.9%) ranked first in the ranking of publications, followed by Materials Science Multidisciplinary (26.5%) in the period 1999–2020.

When the disciplinary network was constructed based on the co-occurrence of disciplines of the publications, the ranking of the important and marginal disciplines in 1999–2020 were noted. The WoS categories of Materials Science Multidisciplinary, Chemistry Physical, Physics Applied, and Nanoscience Nanotechnology were in the core position of the network, and their interactive relationship was closer with others in the network [66]. The network density reflects the closeness of the association between nodes [43]. The network density (0.147) in 1999–2020 was relatively low, suggesting that the overall network association was not strong. The possible reason is that the speed of interdisciplinary development process in the field of natural sciences is slower than that in the field of sociology. As an emerging scientific field, the research period is also relatively short, and the interdisciplinary system has not been fully formed. The details of the whole network demonstrated how things change. On the one hand, the number of discipline interaction and links showed increasing trends. On the other hand, the network density, network centrality, and clustering coefficient decreased gradually. This movement trends were expected given that interdisciplinary plays an important role in the scientific research field with the deepening of academic research.

### 5.2 What are the key disciplines in the scientific field

Comprehensive experiments via node degree analysis indicated the leading disciplines in predicting the academic effect in the network. Superior disciplines were identified and presented. Considering Chemistry, the degree centrality and between centrality of Chemistry Physical ranked the first place, while the closeness centrality of Polymer Science was the biggest. The results suggested that the ability of direct connection of Chemistry Physical was the strongest, and it was close to the center of the cooperation network and influenced the whole network. The discipline with high degree of centrality promotes knowledge diffusion, knowledge sharing, and knowledge innovation in the network interaction, thus improving the efficiency of the whole scientific research cooperation network [67]. Chemistry Physical also played an important role in the mediating position as the main channel for the correlation to other disciplines. Polymer Science showed influence on the other discipline nodes through network structure. The degree centrality, between centrality, and closeness centrality of Materials Science Multidisciplinary were the largest, thus playing an important role in direct connection, mediating channel, and exerting influence in the network. Physics and Physics Condensed Matter, as Materials Science Multidisciplinary, play an important role in the above three functions.

The changes in discipline influence in the network were worth noting. Chemistry Physical continued to play an important role as the major leader in the field from 1999 to 2020. This finding shed light on the fact that more scientific collaboration is needed to maintain a good status. However, some disciplines underwent significant changes in recent years. The influence of Physics Condensed Matter increased obviously in 2020. On the contrary, Environmental Sciences and Physics Atomic Molecular Chemical became weak. With the deepening of scientific collaboration, the changes showed the current IDR trends and hotspots in the field. Therefore, this analysis showed that in the field of PHE, the researchers take Chemistry, Material, and Physical Science as the core subjects and gradually extend to other fields.

Compared with previous studies [68], many other new disciplines, particularly Environment and Medicine Science, have emerged as new key disciplines in this network in photocatalysis. The emerging disciplines have taken advantage of scientific collaborations and enhanced their visibility in scientific network. These new disciplines have started to connect with other disciplines. The two main reasons for the change are as follows: first, the neighboring disciplines tend to form cross cooperation in the field of scientific research [21]; second, the effect of participating disciplines should be based on scientific merit. A new subject knowledge may be needed to solve a new problem in a new field [69]. In the future, photocatalytic technology could be applied in life science, biology, and engineering to solve problems related to life science and medicine.

### 5.3 What are the dynamics of discipline relationship

Considering the dynamics of discipline cluster, blockmodeling analysis showed that the scales of discipline groups gradually expanded. The scale of each cluster included one or two disciplines in 1999 (Table 5), and the level of clustering was very low. Most clusters were only one discipline, that is, Chemistry, indicating not too many disciplines to conduct interaction. The scale was up to 9 in 2020 (Table 6). This dynamic suggested that the number of interactive disciplines increased greatly from 1999 to 2020. The research on PHE extended from one single discipline of Chemistry to more disciplines, including Physics, Bioengineering, and Chemistry.

The dynamics of disciplinary interaction was also explored. The result indicated that interaction relationships between different disciplines tended to be more complex than expected. The relationship between disciplines was considerably simple and weak in 1999 (Fig 5A), whereas the interaction relationship became complex and strong whether it was inter- or intra-discipline group. The result supported the previous findings that scholars have an obvious trend of collaboration in IDR and network influence [70, 71]. The above phenomenon confirmed that forming interaction within a cluster of the disciplines is easier. Given the distance and close relationship in different clusters of the disciplines, a certain difference order pattern was formed in the academic research collaboration network [72].

In addition, the dynamics of interdisciplinary collaboration suggested that the scale of the core network gradually expanded, the trend of various disciplines gathering to the network center increased, and the interaction became more complex. That is to say, the ability of different disciplines, including leaders and participators, to occupy resources were enhanced and the influence of a single discipline on the overall cooperation network declined.

In general, interdisciplinary collaboration plays an increasingly important role in photocatalytic research. A strong trend of interdisciplinary collaboration and interaction occurs in the field. In particular, the trend of interdisciplinary development was more obvious in the emerging science fields.

### 5.4 Research significance

This study focuses on the disciplinary interactions on scientific collaboration network in PHE, with theoretical and practical significance. From a theoretical perspective, a clear disciplinary relationship research path was found in the interdisciplinary scientific collaboration in a research field. The analytical path is a general method that could be used to examine the relationship about the authors, the countries, and so on. It is also applicable for evaluating the leader in the network, such as to identify the academic influence of authors by analyzing the co-authorship and the academic influence of scientific journals by analyzing the co-occurrence of periodicals. So, this study enriched the interdisciplinary cooperative research.

In practice, the results could help accurately identify the interdisciplinary collaboration and provide a reference for scientists in related fields to carry out interdisciplinary collaboration. Given the phenomena of interdisciplinary integration in the deepening of scientific research, interdisciplinary scientific collaborations shall be encouraged to create more chances to generate high scientific innovation. On the one hand, the disciplines located in the networks’ periphery have few connections with multiple other disciplines. Active policy support should be carried out to these periphery disciplines. As the dynamics of the networks may change their position, they could have more new collaborations with other disciplines and an important influence in the networks. On the other hand, as the result of discipline clustering, discipline relationship is more easily formed in its neighboring disciplines. Due to the higher effect of disciplinary boundaries, some disciplines are not inclined to achieve collaborative relationships. Policy makers should provide access to relevant knowledge sources and utilize joint funding schemes and academic exchanges to effectively promote interdisciplinary integration in the world.

## 6 Conclusions and future research

In this study, the network structure characteristics, key disciplines, and dynamic trend of disciplinary interactive relationships in the research field of PHE were examined on the basis of SNA to address the disciplinary interactions in scientific collaboration network. The current research state and evaluation of discipline interaction in an emerging research field were obtained. The research path also established a new concept of discipline relationship in IDR.

In the last 20 years, when scientific studies were constantly breaking through the boundaries of traditional disciplines, integrating the knowledge of multidisciplinary fields and interdisciplinary collaboration was the best method to obtain the solution to scientific and technological problems in the world. Owing to complex social networks, the principal characteristics and dynamics of the interactive relationship between disciplines in the non-reported territory of PHE were determined.

Observation of the tendency of co-authorship publications in the field showed that the interactive collaboration among disciplines moved rapidly from 1999 to 2020, especially after 2010. The topology structure is considered to be becoming increasingly complex, which benefits from the solid combination of multi-discipline knowledges. The intensity of disciplinary interactive relationships is also significantly increasing. In the network, Chemistry Physical, as the key discipline, plays an important role, and it has a strong direct interaction relationship with others all the time. With the temporal evolution, more peripheral disciplines participate and have more interactive relationships in the network. All the disciplines are clustered into different subject groups in accordance with relationship. The scopes of disciplinary groups are expanded, and the interactive relationship between inter- and intra-discipline groups tends to be strengthened and complex.

However, on the literature data of WoS database were collected in this study. Although the platform covers a wide range of literature and provides meaningful data, some papers related to the scientific field may be not covered. This limitation may have some effect on the accuracy of research. Moreover, scientific collaboration may take place in patent collaboration, project fund collaboration, or virtual community interaction collaboration with the deepening of collaboration [73]. With the development of virtual network, scientific cooperation has become more frequent and diversified [74].

Therefore, future research could explore the interdisciplinary phenomena from different perspectives, such as selecting academic social network to analyze the utilization behavior of users in different disciplines. These are helpful to promote the quality of research in interdisciplinary study and provide a potential guideline for scientific researchers.
